# Clinical impact and quality of randomized controlled trials involving interventions evaluating artificial intelligence prediction tools: a systematic review

**DOI:** 10.1038/s41746-021-00524-2

**Published:** 2021-10-28

**Authors:** Qian Zhou, Zhi-hang Chen, Yi-heng Cao, Sui Peng

**Affiliations:** 1 Department of Medical Statistics, Clinical Trials Unit, The First Affiliated Hospital of Sun Yat-sen University, 58 Zhong Shan Er Road, 510080 Guangzhou, China; 2 Department of Liver Surgery, The First Affiliated Hospital, Sun Yat-sen University, 510080 Guangzhou, China; 3 Clinical Trials Unit, The First Affiliated Hospital, Sun Yat-sen University, 510080 Guangzhou, China; 4 Department of Gastroenterology, The First Affiliated Hospital, Sun Yat-sen University, 510080 Guangzhou, China

**Keywords:** Randomized controlled trials, Colon cancer, Predictive markers

## Abstract

The evidence of the impact of traditional statistical (TS) and artificial intelligence (AI) tool interventions in clinical practice was limited. This study aimed to investigate the clinical impact and quality of randomized controlled trials (RCTs) involving interventions evaluating TS, machine learning (ML), and deep learning (DL) prediction tools. A systematic review on PubMed was conducted to identify RCTs involving TS/ML/DL tool interventions in the past decade. A total of 65 RCTs from 26,082 records were included. A majority of them had model development studies and generally good performance was achieved. The function of TS and ML tools in the RCTs mainly included assistive treatment decisions, assistive diagnosis, and risk stratification, but DL trials were only conducted for assistive diagnosis. Nearly two-fifths of the trial interventions showed no clinical benefit compared to standard care. Though DL and ML interventions achieved higher rates of positive results than TS in the RCTs, in trials with low risk of bias (17/65) the advantage of DL to TS was reduced while the advantage of ML to TS disappeared. The current applications of DL were not yet fully spread performed in medicine. It is predictable that DL will integrate more complex clinical problems than ML and TS tools in the future. Therefore, rigorous studies are required before the clinical application of these tools.

## Introduction

An abundance of prediction tools in medicine has been developed and validated to support health decision-making. Prediction tools usually use several predictors to estimate the probability of individuals’ present disease or predict specific situations or events in the future^1–3^. Conventionally, prediction tools are constructed by statistical regression models based on structured patients’ clinical data^4,5^. The recent development of computer technology facilitates the application of machine learning (ML) and even deep learning (DL) algorithms which is a subset of ML in the establishment of prediction tools^6,7^. In contrast to conventional prediction tools, ML- or DL-based prediction tools which are both subsets of artificial intelligence (AI) technology usually use data with high-dimensional features, medical images, or even videos to develop models^8–13^. Many observational studies of model development and validation showed that ML prediction tools performed better than traditional statistical (TS) models in the prediction of disease diagnosis and prognosis by showing higher values of area under the receiver operating characteristic curve (AUC) or accuracy^14–17^. Others found that DL models outperformed standard ML^18,19^. Some of the DL prediction tools have achieved expertise level of diagnostic accuracy in several aspects of diseases^20,21^. Many reports claimed that a well-developed AI prediction tool with adequate performance could assist or even replace clinicians in treatment strategy making for patients' care^11^.

However, some observational studies found that ML prediction algorithms did not outperform TS models for binary outcomes^22–24^, while DL did not always perform better than ML in model development and/or validation studies^25^. More importantly, the clinical effectiveness of these prediction tools based on both traditional and advanced technology for clinical application remains controversial^2,11,22,26,27^. Randomized controlled trials (RCTs) are considered to be the gold standard to establish whether using a prediction tool provides an improvement in the management of patients compared to not using the tool^26,28–31^. This kind of design played an important role in providing high-quality evidence in evidence-based medicine in the past decades^32^. In prediction model research, more and more RCTs involving interventions evaluating TS, ML, and DL tools were published to evaluate the efficacy of a prediction model compared to clinical standard care. The primary outcomes of these RCTs evaluating prediction tools were not patient outcomes which were often difficult to change but other outcomes such as decision-making^33,34^, behavior change^35^, cost-effectiveness^36^ etc. Some of these studies showed that prediction models did not show good clinical benefit in clinical application level^37^. Previous studies have reviewed RCTs evaluating AI interventions in digital health and medical decision support systems, suggesting that the evidence of the effectiveness of AI interventions is limited and contradictory, and their quality is variable^11,38,39^. However, the included studies in these reviews were of small number and in specific fields, and little quantitative analysis was made.

With the increasing number of RCTs evaluating the clinical effectiveness of AI tools recently, concerns about study design and reporting have been raised as well. The Consolidated Standards of Reporting Trials (CONSORT) statement is a 37-item checklist for reporting randomized trials and is widely used in medical research^40^. With the growing recognition of rigorous evaluation for reporting AI trials, the CONSORT statement was planned to adapt to account for specific considerations for AI interventional studies^41,42^. The CONSORT-AI extension has been published recently, which is a 37-item checklist for reporting randomized trials evaluating AI interventions but included 14 new items that are specific for AI interventional trials^42^. Therefore, understanding the clinical effectiveness and quality of these RCTs can provide a reference for more such studies in the future, and vice versa, it may inform future research in model development and application in the early phases of model construction as well.

In this review, we aimed to conduct research of published literature of RCTs involving interventions of traditional statistical or artificial intelligence (TS/AI) prediction tools. First, the quality of these RCTs was evaluated through the Cochrane risk-of-bias tool^43^. Second, the clinical effectiveness of these prediction tools was evaluated according to the main findings of the trial and compared with its previous observational studies of model development and validation.

## Methods

### Study design

This study was a cross-sectional survey on RCTs involving traditional statistical or artificial intelligence (TS/AI) tool interventions in peer-reviewed clinical research journals. The inclusion criteria of RCTs were that (1) the study should be conducted with patients or health professionals, or both, in a clinical setting (population), (2) TS/AI prediction tools were used as a clinical intervention in RCTs (intervention), (3) any types of control group were selected (comparison), (4) quantitative outcomes of the study were presented (outcome), and (5) the article was written in English. The exclusion criteria included (1) studies that were not relevant to interventions using TS/AI tools, (2) reviews and/or meta-analysis, (3) studies of model development and/or validation, (4) observational studies, (5) study protocols or pilot studies, (6) editorial/letters/comments/case report, and (7) studies not in the field of interest. In the current review, we categorized trials into three groups according to the types of intervention tools in clinical practice. They were trials involving interventions evaluating TS, ML, and DL tools, respectively. AI tools included ML and DL algorithms. Though deep learning is a subset of machine learning, the category of ML in the current study did not include DL algorithms. TS models mainly used regression modeling methods, ML included machine learning algorithms, computer-aided diagnosis, Bayesian analysis, and DL used deep convolutional neural networks. No human subjects were involved because the study was mainly a survey of public data and no written informed consent was needed. Studies for prediction tool development and validation of each randomized trial were investigated.

### Search strategy and data sources

We searched PubMed (to Oct 2020) for published papers within the title, abstract, and keywords of the articles. We divided search terms in PubMed into four groups: DL-related terms, ML-related terms, prediction tool-related terms, and terms relating to RCTs. Terms within groups and DL, ML, and prediction tool-related terms were combined with RCTs using the Boolean operator AND, respectively, and the resultant three subgroups were combined using the Boolean operator OR. We referred and modified filters from previous studies^11,44^ to identify AI studies, prediction tools, and RCTs and provided search strategies in Supplementary Note 1.

A search was also conducted in the clinical trial registry website (clinicaltrials.gov, to Oct 2020) using the terms ‘artificial intelligence’, ‘machine learning’, ‘deep learning’ and ‘prediction model/tool’ to identify finished clinical trials for TS/AI interventions. Furthermore, reference lists of each relevant impact analysis study were included to identify possible additional studies. Y.h.C. and Q.Z. independently screened the identified articles following the literature search to minimize selection bias. Any disagreements were resolved by discussion till all investigators reached a consensus.

We screened the abstracts of the candidate articles for inclusion and subsequently read the full text of the articles deemed eligible according to the inclusion criteria. Subsequently, we excluded those ineligible articles and articles not providing sufficient information about the application of TS/AI tools. The studies for prediction tool development and/or validation of each randomized trial were searched according to the descriptions and the citation of the references of the paper. The systematic review was conducted following the Preferred Reporting Items for Systematic Reviews and Meta-Analyses (PRISMA) guidelines^45^. Supplementary Table 1 shows a completed PRISMA checklist.

### Data collection and definition

Data extraction was performed by Y.h.C. and Q.Z. using an Excel spreadsheet (Excel for Windows 2013; Microsoft, Redmond, WA, USA) with the following items for each relevant article: (1) first author; (2) year of publication; (3) type of TS/AI tools (TS, ML, or DL); (4) target of TS/AI tools (assistive diagnosis, risk stratification, assistive treatment decision, or others); (5) algorithms of TS/AI tools; (6) input and output; (7) controls; (8) clinical domain or condition; (9) application setting: inpatient, outpatient, home; (10) performance of the algorithm in model development and/or validation measured by the area under the receiver operating characteristic (AUC) and accuracy, and their 95% confidence intervals; (11) primary outcome of interest: whether it was significantly positive or not, and how the outcome was being used; (12) number of enrolled participants; (13) planned sample size (sufficient or not, defined as the number of enrolled participants larger or equal to the planned number); (14) duration of studies; (15) referenced CONSORT (yes or no); (16) study design and relevant features: masking (open-label, single-blinded or double-blinded), intent-to-treat (ITT) analysis (yes or no), and subgroup analysis (yes or no). For observational studies of model development and/or validation, we exacted data including year of publication, study type (prospective or retrospective), sample size for model development, whether the Transparent Reporting of a multivariable prediction model for Individual Prognosis or Diagnosis [TRIPOD]^46^ was referenced (yes or no), and performance of the algorithm in model development and/or validation measured by AUC and/or accuracy.

Because of the heterogeneity of these trials, the actual effect size for each trial was not able to be synthesized. According to the statistical significance of the primary outcome of interest, we classified a trial as positive if the proposed primary outcome of interest was reached, which means the null hypothesis was rejected, if the 95% confidence intervals excluded the null hypothesis or if the pre-specified target was met. If the primary objective was not stated, a trial was considered positive if the TS/AI tool was superior to the specified control or standard treatment. In describing the TS/AI interventions, the number of predictors, the outcome the algorithm was predicting, and how the outcome was being used to make a decision in the trial were documented.

### Methodological and reporting quality assessment

The quality of each article was independently performed by two reviewers (Q.Z. and Y.h.C.). RCTs were assessed according to the Cochrane Collaboration’s tool for risk of bias^43^. Checked risk of bias and data for published trials were presented. The quality of reporting was assessed according to whether the CONSORT statement was referenced or not^40^. We did not use the CONSORT-AI extension as a reference because the included articles were published before the statement extension was released.

### Statistical analysis

Continuous variables were presented as mean ± standard deviation (SD) or median (interquartile range, IQR), as appropriate, and categorical variables as numbers and percentages. Comparisons between two groups were made using *t* test or Mann−Whitney *U* test for continuous variables and Fisher’s exact test for categorical variables because of small sample size. Subgroup analysis was performed according to the risk of bias of trials. *P* value < 0.05 was considered statistically significant. All statistical analyses and plots were performed using the R version 3.6.0 software (Bell Laboratories, Murray Hill, NJ; https://cran.r-project.org/bin/windows/base/old/3.6.0/).

## Results

### General characteristics

**Fig. 1:**
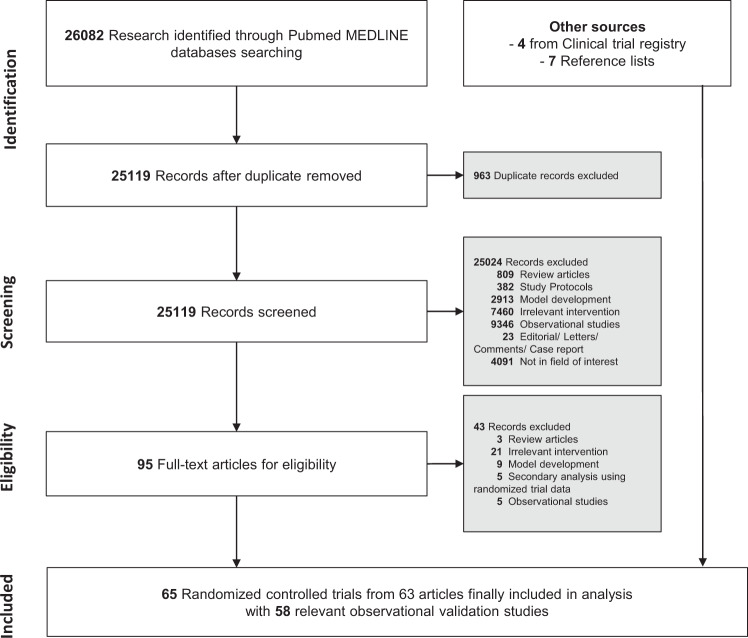
Flowchart of the study. Published trials were searched on PubMed. Clinical trial registry and references in the full-text articles for eligibility were also checked to include potentially relevant trials. Clinical trial registry was the clinicaltrial.gov registry website. The observational studies for tool development and/or validation were searched according to the descriptions and the references of the clinical trial paper.

We screened 26,082 records through PubMed and the registry website from Jan 2010 to Oct 2020, and included 65 trials from 63 articles in the final review and analysis (Fig. 1). There were two articles including two trials conducted in a different population or clinical settings. As we mainly focused on the quality and effectiveness of the studies, we included all the trials separately in each article. A list of included RCTs is shown in Supplementary Table 2.

**Fig. 2:**
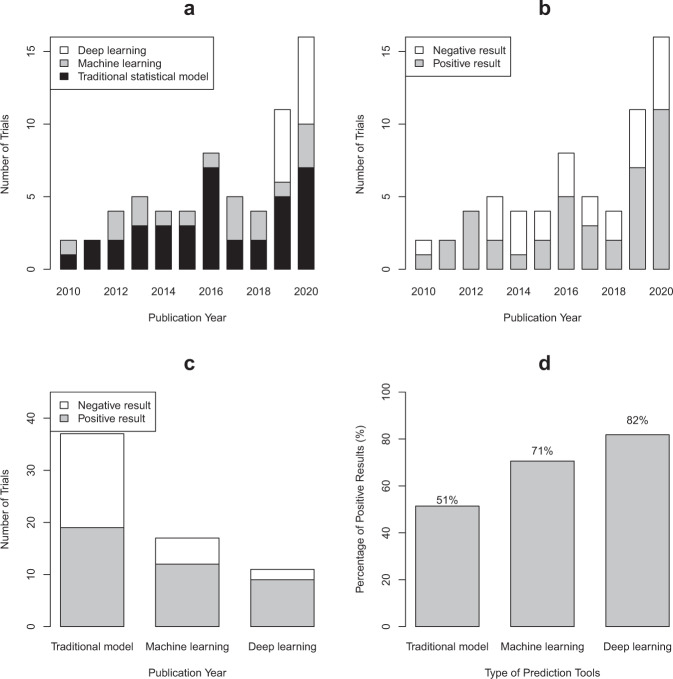
Distribution of the number of trials and percentage of trials with positive results. **a** The trend of published randomized controlled trials involving traditional statistical and artificial intelligence prediction tool interventions with years; **b** the trend of the number of trials with positive and negative results; **c** number of trials with positive results by three types of prediction tools; **d** percentage of trials with positive results by three types of prediction tools.

Of the 65 RCTs, 67.7% were published in 2016 or later. RCTs evaluating DL interventions emerged in 2019. The number of published trials increased over time, with 11 (16.9%), 17 (26.2%), and 37 (56.9%) trials involving DL, ML, and TS prediction tools, respectively (Fig. 2a). Most RCTs did an open-label (75.4%) randomized controlled superiority design (73.8%) with 1:1 allocation ratio (84.6%), and recruited at a single center (50.8%) over a median duration of 12 months for a median sample size of 435 (IQR: 192, 999). The function of these tools in clinical practice included assistive treatment decision (53.8%), assistive diagnosis (24.6%), risk stratification (18.5%), and others (3.1%). The top three covered conditions were acute disease (29.2%), chronic disease not including cancer (27.7%), and cancer (16.9%) (Table 1).

**Table 1:** General characteristics of the 65 randomized controlled trials.

Variables	Levels	Total (*n* = 65)
Results (%)	Negative	25 (38.5)
	Positive	40 (61.5)
Duration of study (*n*^a^ = 59, months, median [IQR])		12 [6, 24]
Sample size (median [IQR])		435 [192, 999]
Sample size estimation (%)	Larger or equal than expected	37 (56.9)
	Less than expected	7 (10.8)
	Not performed	21 (32.3)
Publication year (%)	2010–2015	21 (32.3)
	2016–2020	44 (67.7)
Study design (%)	RCT superiority (individualized)	48 (73.8)
	RCT superiority with crossover (individualized)	1 (1.5)
	RCT non-inferiority (individualized)	2 (3.1)
	Clustered RCT superiority (clustered)	7 (10.8)
	Stepped-wedge design (clustered)	7 (10.8)
Allocation ratio (%)	1:1 parallel	55 (84.6)
	Others	10 (15.4)
Masking (%)	Open-label	49 (75.4)
	Single-blinded	12 (18.5)
	Double-blinded	4 (6.2)
Centers (%)	Single	33 (50.8)
	Multi	32 (49.2)
Disease category (%)	Cancer	11 (16.9)
	Chronic disease not included cancer	18 (27.7)
	Acute disease	19 (29.2)
	Primary care	9 (13.8)
	Others	8 (12.3)
Types of algorithms (%)	Traditional statistical model	37 (56.9)
	Machine learning	17 (26.2)
	Deep learning	11 (16.9)
Prediction tools function (%)	Assistive treatment decision	35 (53.8)
	Assistive diagnosis	16 (24.6)
	Risk stratification	12 (18.5)
	Others	2 (3.1)
Referenced CONSORT (%)	No	47 (72.3)
	Yes	18 (27.7)
Intent-to-treat analysis (%)	No	39 (60.0)
	Yes	26 (40.0)
Study protocol available	No	49 (75.4)
	Yes	16 (24.6)
Model development (%)	No	7 (10.8)
	Yes—independent publication	49 (75.4)
	Yes—published in the same article with RCT	9 (13.8)
Internal validation (%)	No	23 (35.4)
	Yes	42 (64.6)
External validation (%)	No	25 (38.5)
	Yes	40 (61.5)
AUC in model development (*n*^a^ = 21, median [IQR])		0.81 [0.75, 0.90]
AUC in internal validation (*n*^a^ = 18, median [IQR])		0.78 [0.73, 0.78]
AUC in external validation (*n*^a^ = 20, median [IQR])		0.83 [0.79, 0.97]

*IQR* interquartile range, *AUC* area under the receiver operating characteristic curve.

^a^Available numbers used for description

### Quality of reporting and risk of bias assessment for trials involving interventions evaluating traditional statistical and artificial intelligence prediction models

**Fig. 3:**
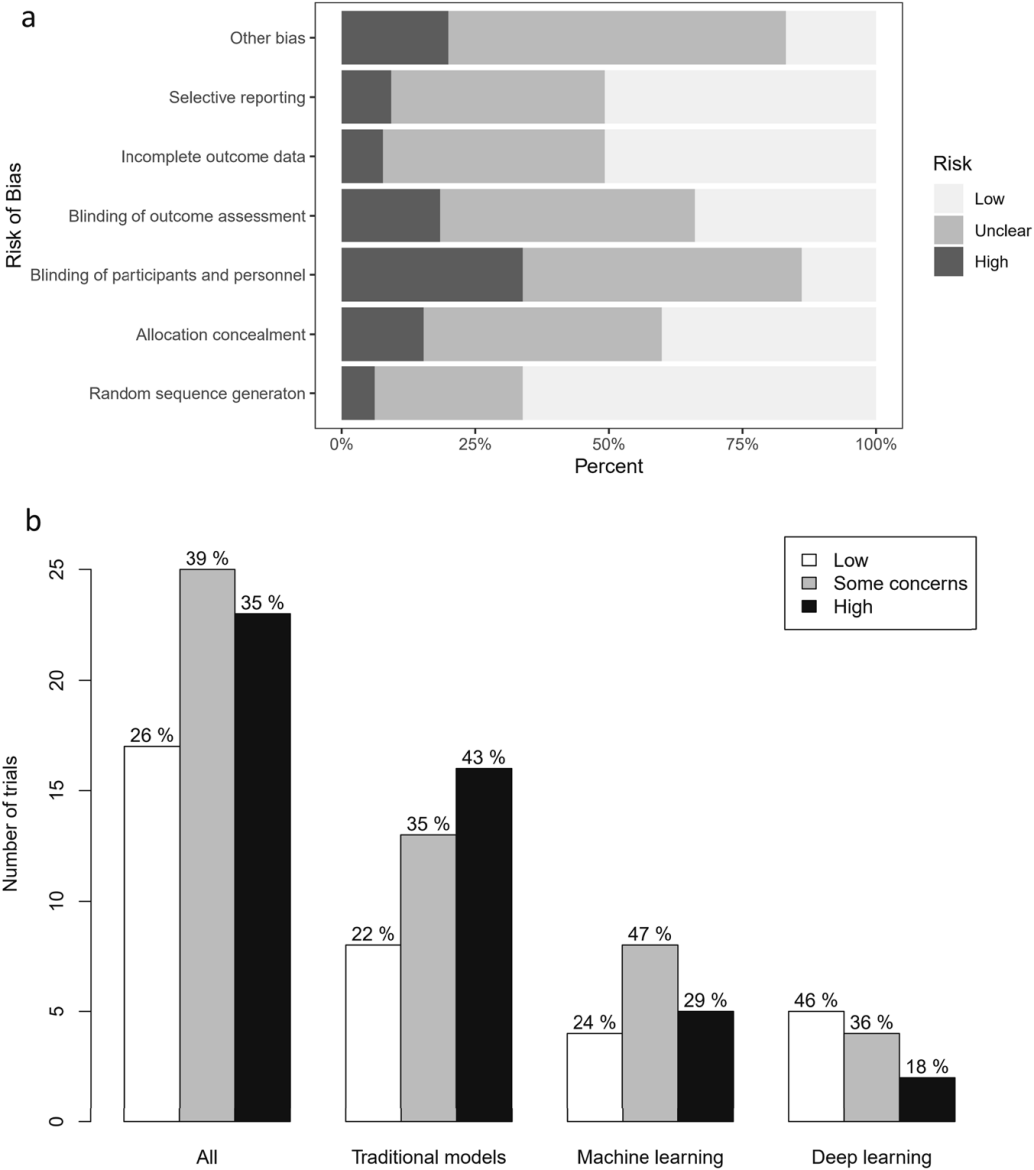
Risk of bias assessment. **a** The distributions of risk of bias by each domain; **b** the distributions of the overall risk of bias for all trials and for traditional statistical, machine learning, and deep learning tools, respectively.

The distributions of risk of bias by each domain and the overall risk of bias of all trials and by types of tools are depicted in Fig. 3a, b.

Blinding of participants and personnel, other bias, blinding of outcome assessment showed a more frequent high risk of bias than other domains. Seventeen trials (26.2%) were considered to have an overall low risk of bias, 38.5% some concerns, and 35.4% overall high risk. When stratified by types of tools, more (46%) trials evaluating DL tools showed a low risk of bias and less (18%) showed a high risk of bias, and nearly half of the trials (43%) involving TS as interventions showed an overall high risk of bias, but the difference was not statistically significant among the three groups (*P* = 0.395). Nearly three quarters (72.3%) did not reference the CONSORT statement in reporting of the trials. Nineteen RCTs (32.3%) did not perform sample size pre-estimation and seven trials (10.8%) recruited subjects less than expected. Most (60.3%) of the trials did not use or mention intent-to-treat analysis. All trials were registered in advance, but study protocols were not available in most of the trials (75.4%) (Table 1).

### AI tool intervention and its performance in both observational model development and/or validation study and randomized controlled trial

All studies reported the *P* value from a comparison of the primary outcome of intervention and control groups. Compared with the control group, two-fifths (38.5%) of the trials showed no benefit as no statistically significant difference for the primary outcome while 61.5% showed positive results (Table 1). The number of trials with positive results for primary outcome increased with time, but the number of trials with negative results did not change much (Fig. 2b).

We found that 58 trials (89%) had model development studies. Nine of them were published in the same paper with the RCT, and 49 were published independently before the trial. Model development studies were not found in seven trials (10.8%), five from TS and two from ML. Most (64.6%; 42/65) of the trials had internal validation using methods of cross-validation, bootstrapping, random split, and split-sample by time point, and 61.5% (40/65) had external validation which was defined as making validation in independent datasets before the corresponding RCT was conducted. These observational studies had a median sample size of 1392 (IQR: 192, 10,356). Most (63.1%) of them were retrospective studies and some (16.9%) were prospective. Only two studies were reported according to the TRIPOD guidance which has been widely used for reporting clinical prediction models. In terms of model performance, 21 of them reported a median AUC of 0.81 (IQR: 0.75, 0.90) in model development. The median AUCs were 0.78 (IQR: 0.73, 0.88; *n* = 18), 0.83 (IQR: 0.79, 0.97; *n* = 11) in internal and external validation, respectively. Data of AUC were not available mainly because of different methods for assessing model performance or not being reported in the final paper. More information on observational studies is shown in Supplementary Table 3.

Table 2 and Supplementary Table 4 show the brief descriptions of 28 RCTs involving deep learning and machine learning interventions in terms of conditions, sample size, tools for intervention and control group, algorithms, the input and output of the tool, how the output is being used in clinical settings, trial outcomes, the gold standard of the outcome, trial findings. Most DL tools were developed for the diagnosis of gastroenterological oncology, but they showed slight differences in the tool outputs. The control group in these trials was routine clinical examination, such as colonoscopies, esophagogastroduodenoscopy. In order to avoid potential operational bias, one DL trial used a sham AI system as control, so that a double-masked design could be performed. Most ML tools exhibited assistive function of patient management and treatment decision for chronic disease.

**Table 2:** Procedures of predictive tool interventions in the 11 randomized controlled trials involving interventions evaluating deep learning tools.

Reference	Conditions	Sample size	Tools for intervention	Control	Algorithms	Tool function	Tool input	Tool output	How the output being used in clinical settings	Trial primary outcomes	Gold standard	Trial findings
	Upper gastrointestinal lesions	437	Routine EGD examination stratified by three types with the assistance of ENDOANGEL AI system	Routine EGD examination stratified by three types without AI	DCNN (VGG-16)	Assistive diagnosis	EGD images	A virtual stomach model monitoring blind spots; timing; scoring and grading	Experts referenced AI output to make EGD examination and monitor blind spots.	Mean blind spot rate	Experts	Positive
	Childhood cataracts	700	CC-cruiser web diagnosis platform	Regular ophthalmic diagnosis	DCNN (ImageNet)	Assistive diagnosis	Ocular images from slit-lamp photography	Diagnosis outcome; comprehensive evaluation; treatment recommendation	AI made diagnosis independently, and its results would be comparted with experts and not impact clinical decision making.	Accuracy of diagnosis	Experts	Negative
	Colorectal cancer	659	Routine colonoscopies with the assistance of an AI automatic quality control system	Routine colonoscopies	DCNN (AlexNet, ZFNet, YOLO V2)	Assistive diagnosis	Colonoscopy images	Location of colorectal polyps; timing; reminding retest and clean	Endoscopists referenced AI output to make endoscopic examination and report of polyps and adenomas.	Adenoma detection rate	Pathology	Positive
	Colorectal cancer	1058	Routine colonoscopies with the assistance of an automatic polyp detection system	Routine colonoscopies	Deep learning architecture	Assistive diagnosis	Colonoscopy images	Location of polyps; alarming	Endoscopists were required to check every polyp location detected by the system and report of polyps and adenomas.	Adenoma detection rate	Pathology	Positive
	Upper gastrointestinal lesions	303	Routine EGD examination with the assistance of WISENSE AI system	Routine EGD examination	DCNN (VGG-16 and DenseNet)	Assistive diagnosis	EGD images	A virtual stomach model monitoring blind spots; timing; scoring and grading; extracting frames with the highest confidence	Experts referenced AI output to make EGD examination and monitor blind spots.	Mean blind spot rate	Experts	Positive
	Colorectal cancer	704	ENDOANGEL-assisted routine colonoscopy	Routine colonoscopy	DCNN and perceptual hash algorithms (VGG-16)	Assistive diagnosis	Colonoscopy images	Timing; safe, alarm, and dangerous ranges of withdrawal speed for real-time monitoring; slipping warning	Operating endoscopists referenced AI output to make endoscopic examination and report of polyps and adenomas.	Adenoma detection rate	Pathology	Positive
Liu (2020)^42^	Colorectal cancer	1026	Routine colonoscopy with CADe assistance	Routine colonoscopy	DCNN-3D	Assistive diagnosis	Colonoscopy images	The probability of polyps in each frame; lesions alarming	Endoscopists focused mainly on the main monitor during the examination process, and a voice alarm prompted them to view the system monitor to check the location of each polyp detected by the system.	Detection rate of polyps and adenomas	Pathology	Positive
	Colorectal cancer	157	AI-assisted colonoscopy	Traditional colonoscopy	CNN (YOLO)	Assistive diagnosis	Colonoscopy images	Location of polyps	Endoscopists referenced AI output to make endoscopic examination and report of polyps.	Polyp detection rate	Not reported	Positive
Repici (2020)^62^	Colorectal cancer	685	High-definition colonoscopies with the AI-based CADe system	Routine colonoscopy	CNN	Assistive diagnosis	Colonoscopy images	Location of polys	Endoscopists referenced AI output to make endoscopic examination and report of polyps and adenomas.	Adenoma detection rate	Pathology	Positive
Wang (2020)^61^	Colorectal cancer	962	White light colonoscopy with assistance from the CADe system	White light colonoscopy with assistance from a sham system	Deep learning architecture	Assistive diagnosis	Colonoscopy images	Location of polyps; alarming	Endoscopists were required to check every polyp location detected by the system and report of polyps and adenomas.	Adenoma detection rate	Pathology	Positive
Blomberg (2021)^58^	Out-of-hospital cardiac arrest (OHCA)	5242	Normal protocols with alert	Normal protocols without alert	Speech recognition using deep neural networks	Assistive diagnosis	Emergency calls	OHCA Alert	Dispatchers in the intervention group were alerted when the machine learning model identified out-of-hospital cardiac arrest.	The rate of dispatcher recognition of subsequently confirmed OHCA	Danish Cardiac Arrest Registry	Negative

*AI* artificial intelligence, *DL* tools using deep learning algorithms, *ML* tools using machine learning algorithms, *CNN* convolutional neural networks, *DCNN* deep convolutional neural networks, *CADe* computer-aided detection, *EGD* esophagogastroduodenoscopy, *OHCA* out-of-hospital cardiac arrest.

### Comparisons among trials involving interventions of traditional statistical, machine learning, and deep learning tools

The intervention tools were classified into three categories according to their types of algorithms (Table 3). Trials involving ML and DL tool interventions took less time duration than TS interventions (7 vs 6 vs 18 months, *P* = 0.005). The median sample size of trials evaluating the TS, ML, and DL tools was 435 [IQR: 194, 999], 258 [IQR: 90, 537], and 700 [IQR: 548, 994], respectively, but no statistical significance (*P* = 0.122). These models were implemented in different clinical settings (*P* = 0.015). A majority of DL interventions were for inpatients and used non-quantitative clinical data such as computed tomography (CT), magnetic resonance imaging (MRI), slit-lamp photography, colonoscopy, or esophagogastroduodenoscopy. The proportions of disease categories and the function of prediction models were not consistent among the three types of interventions (both *P* < 0.001). Trials evaluating DL interventions were more likely conducted in cancer research, such as colorectal cancer, upper gastrointestinal cancer, and all of them were for the purpose of disease assistive diagnosis. ML interventions were more frequently used in chronic diseases, not including cancer such as obesity, work disability, anemia, and so on, and a majority of them were used for assistive treatment decisions and some for assistive diagnosis. While TS tool interventions were more in acute diseases such as mechanical acute small bowel obstruction, acute heart failure, treatment decisions in intensive care units, and their purposes were diverse including assistive treatment decision, risk stratification, and assistive diagnosis.

**Table 3:** Comparisons among trials involving traditional statistical, machine learning and deep learning predictive tool interventions.

Variables	Levels	TS (*n* = 37)	ML (*n* = 17)	DL (*n* = 11)	*P* value
Duration of study (*n* = 59, months, median [IQR])		17 [8, 32]	7 [4, 19]	6 [4, 9]	0.005
Sample size (median [IQR])		435 [194, 999]	258 [90, 537]	700 [548, 994]	0.122
Clinical settings (%)	Outpatients	19 (51.4)	6 (35.3)	1 (9.1)	0.015
	Inpatients	17 (45.9)	8 (47.1)	10 (90.9)	
	Home	1 (2.7)	3 (17.6)	0 (0.0)	
Publication year (%)	2010–2015	14 (37.8)	7 (41.2)	0 (0.0)	0.041
	2016–2020	23 (62.2)	10 (58.8)	11 (100.0)	
Model input (%)	Clinical quantitative data	36 (97.3)	16 (94.1)	0 (0.0)	<0.001
	Images or videos	1 (2.7)	0 (0.0)	10 (90.9)	
	Natural language	0 (0.0)	1 (5.9)	1 (9.1)	
Disease category (%)	Cancer	2 (5.4)	0 (0.0)	9 (81.8)	<0.001
	Chronic disease	4 (10.8)	13 (76.5)	1 (9.1)	
	Acute disease	16 (43.2)	2 (11.8)	1 (9.1)	
	Primary care	9 (24.3)	0 (0.0)	0 (0.0)	
	Others	6 (16.2)	2 (11.8)	0 (0.0)	
Prediction tools function (%)	Assistive diagnosis	3 (8.1)	2 (11.8)	11 (100.0)	<0.001
	Risk stratification	11 (29.7)	1 (5.9)	0 (0.0)	
	Assistive treatment decision	22 (59.5)	13 (76.5)	0 (0.0)	
	Others	1 (2.7)	1 (5.9)	0 (0.0)	
Results (%)	Negative	18 (48.6)	5 (29.4)	2 (18.2)	0.136
	Positive	19 (51.4)	12 (70.6)	9 (81.8)	0.044 (*P* for trend)

*TS* randomized controlled trials involving traditional statistical tool as intervention, *ML* randomized controlled trials involving tool using machine learning algorithms not including deep learning as intervention, *DL* randomized controlled trials involving tool using deep learning algorithm as intervention.

**Fig. 4:**
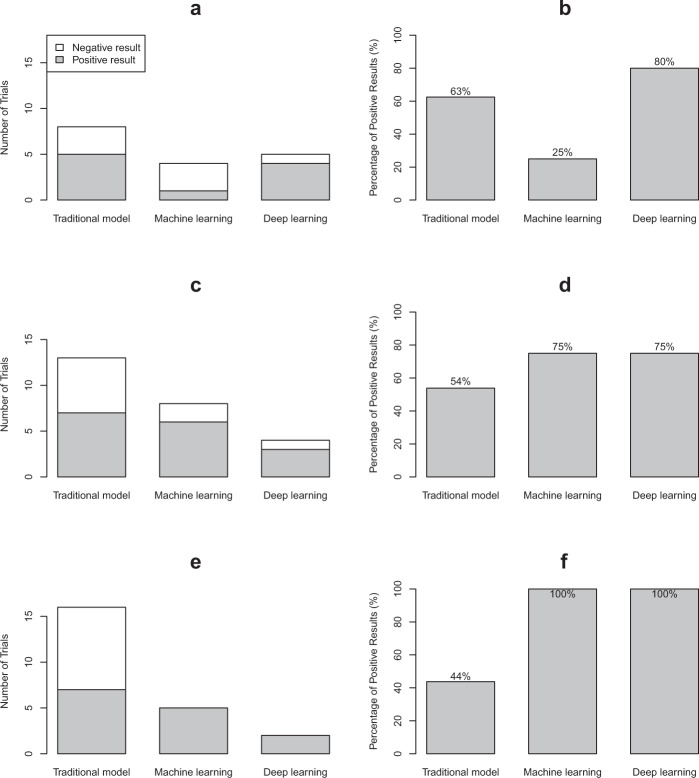
The number of trials and percentage of positive results of three types of tools according to the risk of bias. **a** The number of trials of each type of tool in trials with low risk of bias; **b** the percentage of positive results of each type of tool in trials with low risk of bias; **c** the number of trials of each type of tool in trials with some concerns; **d** the percentage of positive results of each type of tool in trials with some concerns; **e** the number of trials of each type of tool in trials with a high risk of bias; **f** the percentage of positive results of each type of tool in trials with a high risk of bias.

The positive rates of primary analysis were different among trials involving interventions evaluating TS, ML, and DL tools (51.4%, 70.6%, 81.8%, respectively; *P* for Fisher exact test = 0.136, *P* for trend = 0.044) (Fig. 2c, d). However, when we stratified by the risk of bias (low, some concerns, high), the distribution of the positive rate of results was changed (Fig. 4). In trials with low risk of bias, the positive rate of trials involving TS tools increased to 63%, ML tools decreased to 25%, and DL tools remained (80%), but no statistically significant difference was found (*P* for Fisher exact test = 0.374, *P* for trend = 0.660; Fig. 4a, b). Only in the subgroup of high risk of bias, the positive rates were significantly different (TS, ML, DL: 44%, 100%, 100%, *P* for Fisher exact test = 0.035, *P* for trend = 0.019; Fig. 4e, f).

## Discussion

In the current study, we found that the number of RCTs evaluating TS/AI interventions increased with year in the past decade, and trials involving AI tools multiplied in recent 2 years. However, we should be cautious about the clinical application of TS/AI prediction tools before the effectiveness has been proved in rigorous clinical research. This review showed that only a quarter of trials were assessed to be low risk of bias. Consistent with other studies reviewing the quality of RCTs in both general medical fields and in AI^11,38,39,47,48^, the quality of the trials in the current study tended to be suboptimal in the aspects of referenced CONSORT statement, sample size pre-estimation, randomization, masking, and intent-to-treat analysis. In addition, in this cross-sectional survey through published literature of 65 RCTs, two-fifths of TS/AI prediction tools that achieved good performance in observational model development and/or validation studies failed to show clinical benefit for patients compared to routine clinical treatment. DL and ML tools exhibited superiority to TS tools with regard to the percentage of positive results. However, in trials with a low risk of bias, this advantage in DL remained but disappeared in ML. The percentage of positive rate remained in DL trials, and increased in TS trials, but decreased a lot in ML trials.

We focused on RCTs involving interventions evaluating TS/AI prediction tools to make the assessment and evaluation of the effectiveness of the TS/AI-based interventions as well as the quality of these studies. In recent 2 years, a few studies have been published to review the current situation of AI-based interventional studies from RCTs to evaluate its effectiveness, quality, and methodology^11,38,39^. They were reviews including five, eight, and two AI-based RCTs, respectively. Cresswell et al.^38^ selected AI-based RCTs because they thought that, compared with other observational research designs, the risk of bias of RCTs was the lowest. Triantafyllidis et al.^39^ found that digital health intervention involving AI could be useful and effective based on eight RCTs. But five of them were pilot studies or studies of wearable devices, which were inconsistent with the inclusion criteria of the current study. In short, although a small number of previous studies have discussed the situation of AI-based interventional studies in several medical fields, these reviews were unable to make quantitative analysis and conclusions because of their small sample size and less rigorous methodology. Therefore, we made broader criteria for including RCTs to evaluate the clinical effectiveness of AI prediction tools and compared them with TS trials. In the present study, we focused on prospective RCTs not only in medical images but also in other medical fields. Although there are inherent limitations in RCTs^49^, we believe that as a gold standard design, the results from RCTs could help us to understand more about the progress and effectiveness of TS/AI tool interventions.

A total of 65 RCTs were included in the current study, and nearly half of them used AI tools as interventions. We found a moderate proportion of negative results in these trials. This indicated that even achieving good performance in model development and being well-validated, prediction-tool-based interventions might fail to show clinical benefit for patients when compared to routine care in RCTs. No agreement has been reached on how much evidence is needed before a prediction tool could be utilized in clinical work. Some researchers^9^ tried to adopt clinical trial phases for the drug development process to simulate the development process of medical image mining tools. According to their proposed process, a prospective design for validation with more than 100 sample sizes was defined as Phase III. However, the identified eight “Phase III trials” in their study were less evidence to be implemented in clinical settings^50^. Nagendran et al.^11^ reviewed 83 published clinical studies of deep learning in medical imaging diagnosis from 2010 to June 2019 and found that the superiority of AI tools over clinicians was overpromising. But there were only nine prospective deep learning studies and two randomized trials existing in medical imaging, so their conclusions were largely based on retrospective studies. Our study included more AI RCTs than previous reviews and we chose TS trials as comparisons. Through the results of these RCTs, we found that although AI tools showed more percentages of better clinical outcomes than traditional care or routine examination than that of TS tools, in the subgroup of low-risk of bias studies, the rate of a positive result in TS trials increased a lot and was not inferior to that of AI tools. Therefore, we believe that high-quality clinical trial designs, such as RCTs, are still required to assess the effectiveness of TS/AI prediction tools before they are implemented in clinical settings. CONSORT-AI group^41^ has been working on the reporting guideline for clinical trials evaluating AI interventions, which will help evaluate the reporting of these trials.

In addition, a high-quality and rigorous study design was required to conduct RCTs evaluating TS/AI interventions. In our study, the quality of the included trials was variable, which is consistent with several previous reviews^11,38,39^. Principles of trials’ design such as masking, randomization, and allocation concealment, reporting referenced CONSORT statement was not well followed in the included trials. It is worth noting that these issues are not unique to TS/AI trials. In fact, in previous RCTs in general, the quality was found to be well below an acceptable level^47,48^. In addition, the risk of bias assessment showed a low proportion of low risk and a high proportion of high risk of bias in the included trials. In doing the assessment, we focused on randomization and masking. Some of the trials^51–53^ gave the reason why they did not use masking for its difficulty or its nature of the intervention, and some would be not influenced by non-masking^54–56^. Masking was also related to study design (i.e. stepped-wedge cluster RCTs)^57^. Therefore, we did not consider such open-label trials as high risk in the blinding domain of risk of bias assessment after a comprehensive evaluation. Encouragingly, four trials used double-blinded design^58–61^. One of them is a DL trial^61^ to assess the effectiveness of an AI system compared with a sham system so that participants could be blinded to study groups. A sham system was adopted as a control, which was developed from polyp-like non-polyp structure with high sensitivity and zero specificity to detect polyps. This allowed avoiding potential operational bias. Although cluster randomization was recommended to a preferred design by researchers more than 10 years ago^27^, there was a fifth of trials using clustered randomization. In order to compare with human performance or routine clinical treatment, most trial hypotheses were that the prediction tool would show superiority to clinicians. Non-inferiority design (2/65) would be a choice to prove that the performance of the prediction tool is not inferior to expertise^62^.

This study showed that DL tools tended to obtain more positive results compared to ML and TS models. However, RCTs involving DL tools nowadays were conducted in a narrower field of diseases and had simpler targets than that of ML and TS trials. For example, DL prediction tools were mostly for the diagnosis and detection of colorectal cancer. Of note, there were seven trials concerning AI’s application in colonoscopy. This is relevant to the increasing number of published studies on the AI model construction of colonoscopy in recent years^8^. Consistent with other reviews in deep convolutional neural network‐based AI on colonoscopy^63^, AI assistive colonoscopy was promising but still need more application in different population. ML and TS RCTs were conducted in more application scenarios for different purposes, showing great flexibility and uncertainty in results. For example, Bailey et al.^64^ in 2013 used a logistic regression prediction model to make real-time automated alerts for patients every day and send alerts to nurses to signify the risk of transfer to intensive care. Geersing et al.^54^ in 2020 used a Cox regression prediction model to estimate patients’ recurrence risk and then make model-assisted treatment recommendations for patients. These RCTs tried to solve important clinical problems but unfortunately failed. Of note, the percentage of a positive rate of TS trials increased from 51% in all the trials to 63% in the low-risk trials, while ML interventions decreased from 71% in all the trials to 25%. This change after stratifying by the risk of bias was also observed in observational studies for prediction model development which showed that no performance benefit of ML over logistic regression for clinical prediction models^22^. The current applications of AI are not yet fully spread performed in medicine, and in the future, it will integrate more clinical problems like ML and TS tools. This gives us a hint that if AI tools will be used in a wider range of scenarios in medicine in the future, the process may be more complex and results may face more uncertainty.

### Limitations

There were some limitations in the study. Firstly, although comprehensive, our search might have missed some studies that could have been included. In order to validate our search strategy, we specifically paid close attention to trials in high-quality journals and also searched for specific studies or study designs, such as trials for computer decision support, TS/AI trials using a cluster randomized controlled design, and reports of relevant trials on websites. Second, given the heterogeneity of these published trials, no meta-analysis was performed. The current study was a systematic review of trials involving interventions evaluating TS/AI prediction tools, and we analyzed the quality of methodology and risk of bias of the included trials. Third, the trials included in this review were published before the publication of CONSORT-AI extension^42^, so we did not evaluate the reporting of these trials according to the new guidance. We extracted information on whether these trials referenced CONSORT^40^ or not.

### Future work

Based on our study, we made some recommendations for future research.

#### Rigorous trial design such as randomized controlled trial to study evaluating TS/AI tools

This could make the evidence of the performance of a TS/AI tool more reliable before it is used in clinical practice and accelerate clinical translation.

#### Application of CONSORT-AI for reporting

The articles reporting RCTs evaluating TS/AI tools should comply with CONSORT-AI^42^ before publication. This could improve the quality of reporting of RCTs evaluating TS/AI tools.

#### Development of specific tools of evaluating the risk of bias for RCTs evaluating TS/AI tools

Currently, there is no specific standard for the assessment of the risk of bias for RCTs evaluating TS/AI tools. With the rapid development of AI tools, it is urgent to develop a specific tool for evaluating the risk of bias of these studies, which can make the results and conclusions of this kind of trials more convincing.

## Conclusion

Although negative results have been consistently reported in RCTs involving TS/AI prediction tools, an increasing proportion of studies with positive results in DL prediction tool interventions showed promising perspectives. Whereas the current applications of DL tools are not yet fully widely performed in medicine, and in the future, it will integrate more clinical problems like ML and TS tools. However, ML tools in RCTs showed variable results because in trials at low risk of bias, ML tools got a very low rate of positive results compared to the other two kinds of tools, while in trials with a high risk of bias, it performed much better. Therefore, we believe that rigorous trial is necessary to obtain evidence of DL prediction tool interventions. The experience of RCTs involving ML and TS tools indicates that we should be cautious about the effectiveness of DL when applied to more complex clinical problems and long-term interventions. In addition, high-quality RCTs with transparent reporting are needed to evaluate the efficacy of intelligence prediction models in clinical settings. Prediction tools with DL algorithms for clinical decision-making are the future trend and will be used in the treatment needs of millions of people. Using high-quality research to carefully validate the most clinically valuable tools for clinical practice will help reduce the burden on physicians and protect subjects.

## Supplementary information

Supplementary Information

## Data Availability

The data that support the findings of this study are available on reasonable request from the authors. A full list of records identified through database searching are included in the supplementary information.

## Code availability

The codes of the paper are available on reasonable request from the authors.
