# Supplementary material for: Clinical impact and quality of randomized controlled trials involving interventions evaluating artificial intelligence prediction tools: a systematic review
Source: NPJ Digit Med. 2021 Oct 28;4:154. doi: 10.1038/s41746-021-00524-2 (PMC8553754; doi:10.1038/s41746-021-00524-2)
Supplement: Supplementary file 1 — Supplementary Information [file 41746_2021_524_MOESM1_ESM.pdf]

Supplementary Note 1. Search strategies in PubMed (MEDLINE)

We divided search terms into four groups: ①Deep learning (DL)-related terms, ② Machine learning (ML)-related terms, ③ prediction tool-related terms, and ④ terms relating to randomized controlled trials (RCTs). Terms within groups were combined with the Boolean operator OR. DL, ML and prediction tool-related terms were combined with RCTs using the Boolean operator AND, respectively. The resulted three subgroups were combined using the Boolean operator OR. That is (#① AND #④) OR (#② AND #④) OR (#③ AND #④). The search terms of each group were described as follows.

① DL-related terms

((((Artificial intelligence OR artificial intelligent OR Computer-aided OR deep learning OR deep-learning OR deep convolutional neural network* OR radiomics OR (automatical OR automatically OR automatic))))) OR ((((((ensemble[Title/Abstract]) AND (deep[Title/Abstract])) OR ((machine learning[Title]) AND (deep[Title/Abstract]))) OR (((artificial intelligence[Title]) OR (AI[Title/Abstract])) OR (neural network*[Title]))) OR (deep learning[Title/Abstract] OR deep-learning[Title/Abstract] OR reinforcement learning[Title/Abstract] OR reinforcement-learning[Title/Abstract] OR deep neural network*[Title/Abstract] OR deep belief network*[Title/Abstract] OR convolutional neural network*[Title/Abstract] OR recurrent neural network*[Title/Abstract] OR feedforward neural network*[Title/Abstract])) OR (Boltzmann machine*[Title/Abstract] OR long short-term memory[Title/Abstract] OR gated recurrent unit[Title/Abstract] OR rectified linear unit[Title/Abstract] OR autoencoder[Title/Abstract] OR backpropagation[Title/Abstract] OR multilayer perceptron[Title/Abstract] OR convnet[Title/Abstract] OR convolutional learning[Title/Abstract]))

② ML-related terms

(machine learning[tiab]) OR (supervised learning[tiab]) OR (unsupervised learning[tiab]) OR (deep learning[tiab]) OR (artificial Intelligence[tiab]) OR (decision trees[tiab]) OR (Artificial[tiab]) OR (Neural Network[tiab]) OR (CNN[tiab]) OR

(ANN[tiab]) OR (Convolutional Neural Network[tiab]) OR (random forest[tiab]) OR (reinforcement learning[tiab]) OR (gradient boosting[tiab]) OR (computer aided diagnosis[tiab]) OR (CAD[tiab]) OR (computer assisted diagnosis[tiab]) OR (computational analysis[tiab])

③ Prediction tool-related terms

Predicting[tiab] OR Prediction[tiab] OR Predictive[tiab] OR Predictive value of tests[mh] OR Scor*[ti] OR Observer variation[mh]

④ RCTs-related terms

We used a sensitive search strategy that combined the Cochrane Highly Sensitive Search Strategy for RCTs with NOT Publication types

(((trial[ti]) OR (randomly assigned[tiab]) OR ("Clinical Trials as Topic"[Mesh:noexp]) OR (randomized[tiab]) OR (controlled clinical trial[pt]) OR (randomized controlled trial[pt])) NOT (animals[mh] NOT humans[mh])) NOT ((News[ptyp] OR Comment[ptyp] OR Editorial[ptyp] OR Case Reports[ptyp] OR Dictionary[ptyp]) OR (secondary analysis[Title/Abstract]) OR (retrospective[Title/Abstract]) OR (nested case-control[Title]) OR (case-control[Title]) OR (systematic review[Title]) OR (letter to the editor[Title]) OR ("meta-analysis"[Title]) OR ("study protocol"[Title]) OR ("study design"[Title]) OR (rational[Title]))

Supplementary Table 1. PRISMA-2009-Checklist-MS-Word

Section/topic	#	Checklist item	Reported on page #
**TITLE**			
Title	1	Identify the report as a systematic review, meta-analysis, or both.	1
**ABSTRACT**			
Structured summary	2	Provide a structured summary including, as applicable: background; objectives; data sources; study eligibility criteria, participants, and interventions; study appraisal and synthesis methods; results; limitations; conclusions and implications of key findings; systematic review registration number.	3
**INTRODUCTION**			
Rationale	3	Describe the rationale for the review in the context of what is already known.	4-5
Objectives	4	Provide an explicit statement of questions being addressed with reference to participants, interventions, comparisons, outcomes, and study design (PICOS).	6
**METHODS**			
Protocol and	5	Indicate if a review protocol exists, if and where it can be accessed (e.g., Web address), and,	NA

Section/topic	#	Checklist item	Reported on page #
registration		if available, provide registration information including registration number.	
Eligibility criteria	6	Specify study characteristics (e.g., PICOS, length of follow-up) and report characteristics (e.g., years considered, language, publication status) used as criteria for eligibility, giving rationale.	6-7
Information sources	7	Describe all information sources (e.g., databases with dates of coverage, contact with study authors to identify additional studies) in the search and date last searched.	7-9
Search	8	Present full electronic search strategy for at least one database, including any limits used, such that it could be repeated.	7-8 and Supplementary Note 1
Study selection	9	State the process for selecting studies (i.e., screening, eligibility, included in systematic review, and, if applicable, included in the meta-analysis).	Figure 1
Data collection process	10	Describe method of data extraction from reports (e.g., piloted forms, independently, in duplicate) and any processes for obtaining and confirming data from investigators.	9-10
Data items	11	List and define all variables for which data were sought (e.g., PICOS, funding sources) and	9-10

Section/topic	#	Checklist item	Reported on page #
		any assumptions and simplifications made.	
Risk of bias in individual studies	12	Describe methods used for assessing risk of bias of individual studies (including specification of whether this was done at the study or outcome level), and how this information is to be used in any data synthesis.	10
Summary measures	13	State the principal summary measures (e.g., risk ratio, difference in means).	11
Synthesis of results	14	Describe the methods of handling data and combining results of studies, if done, including measures of consistency (e.g., I^2) for each meta-analysis.	NA
Risk of bias across studies	15	Specify any assessment of risk of bias that may affect the cumulative evidence (e.g., publication bias, selective reporting within studies).	11
Additional analyses	16	Describe methods of additional analyses (e.g., sensitivity or subgroup analyses, meta-regression), if done, indicating which were pre-specified.	11
**RESULTS**			
Study selection	17	Give numbers of studies screened, assessed for eligibility, and included in the review, with	11

Section/topic	#	Checklist item	Reported on page #
		reasons for exclusions at each stage, ideally with a flow diagram.	
Study characteristics	18	For each study, present characteristics for which data were extracted (e.g., study size, PICOS, follow-up period) and provide the citations.	11-12
Risk of bias within studies	19	Present data on risk of bias of each study and, if available, any outcome level assessment (see item 12).	12-13
Results of individual studies	20	For all outcomes considered (benefits or harms), present, for each study: (a) simple summary data for each intervention group (b) effect estimates and confidence intervals, ideally with a forest plot.	11-16
Synthesis of results	21	Present results of each meta-analysis done, including confidence intervals and measures of consistency.	NA
Risk of bias across studies	22	Present results of any assessment of risk of bias across studies (see Item 15).	12-13
Additional analysis	23	Give results of additional analyses, if done (e.g., sensitivity or subgroup analyses, meta-regression [see Item 16]).	13-16

Section/topic	#	Checklist item	Reported on page #
**DISCUSSION**			
Summary of evidence	24	Summarize the main findings including the strength of evidence for each main outcome; consider their relevance to key groups (e.g., healthcare providers, users, and policy makers).	16-17
Limitations	25	Discuss limitations at study and outcome level (e.g., risk of bias), and at review-level (e.g., incomplete retrieval of identified research, reporting bias).	21-22
Conclusions	26	Provide a general interpretation of the results in the context of other evidence, and implications for future research.	23
**FUNDING**			
Funding	27	Describe sources of funding for the systematic review and other support (e.g., supply of data); role of funders for the systematic review.	24

Supplementary Table 2. Listing of recruited articles (65 trials from 63 articles)

Index	Articles
1	Brier ME, Gaweda AE, Dailey A, Aronoff GR, Jacobs AA. Randomized trial of model predictive control for improved anemia management. Clin J Am Soc Nephrol. 2010;5(5):814-20.
2	Stiell IG, Clement CM, Grimshaw JM, Brison RJ, Rowe BH, Lee JS, et al. A prospective cluster-randomized trial to implement the Canadian CT Head Rule in emergency departments. CMAJ : Canadian Medical Association journal = journal de l'Association medicale canadienne. 2010;182(14):1527-32.
3	Hill JC, Whitehurst DG, Lewis M, Bryan S, Dunn KM, Foster NE, et al. Comparison of stratified primary care management for low back pain with current best practice (STarT Back): a randomised controlled trial. Lancet. 2011;378(9802):1560-71.
4	Sheridan SL, Draeger LB, Pignone MP, Keyserling TC, Simpson RJ, Jr., Rimer B, et al. A randomized trial of an intervention to improve use and adherence to effective coronary heart disease prevention strategies. BMC health services research. 2011;11:331.
5	Martin CM, Vogel C, Grady D, Zarabzadeh A, Hederman L, Kellett J, et al. Implementation of complex adaptive chronic care: the Patient Journey Record system (PaJR). Journal of Evaluation in Clinical Practice. 2012;18(6):1226-34.
6	Nieuwlaat R, Hubers LM, Spyropoulos AC, Eikelboom JW, Connolly BJ, Van Spall HG, et al. Randomised comparison of a simple warfarin dosing algorithm versus a computerised anticoagulation management system for control of warfarin maintenance therapy. Thrombosis and haemostasis. 2012;108(6):1228-35.
7	Pielmeier U, Rousing ML, Andreassen S, Nielsen BS, Haure P. Decision support for optimized blood glucose control and nutrition in a neurotrauma intensive care unit: preliminary results of clinical advice and prediction accuracy of the Glucosafe system. Journal of clinical monitoring and computing. 2012;26(4):319-28.

Index	Articles
8	Sáenz A, Brito M, Morón I, Torralba A, García-Sanz E, Redondo J. Development and validation of a computer application to aid the physician's decision-making process at the start of and during treatment with insulin in type 2 diabetes: a randomized and controlled trial. Journal of diabetes science and technology. 2012;6(3):581-8.
9	Bailey TC, Chen Y, Mao Y, Lu C, Hackmann G, Micek ST, et al. A trial of a real-time Alert for clinical deterioration in Patients hospitalized on general medical wards. Journal of Hospital Medicine. 2013;8(5):236-42.
10	Finkelstein SM, Lindgren BR, Robiner W, Lindquist R, Hertz M, Carlin BP, et al. A randomized controlled trial comparing health and quality of life of lung transplant recipients following nurse and computer-based triage utilizing home spirometry monitoring. Telemedicine journal and e-health : the official journal of the American Telemedicine Association. 2013;19(12):897-903.
11	Hsu JC, Chen YF, Chung WS, Tan TH, Chen T, Chiang JY. Clinical verification of a clinical decision support system for ventilator weaning. Biomedical engineering online. 2013;12 Suppl 1(Suppl 1):S4.
12	McGinn TG, McCullagh L, Kannry J, Knaus M, Sofianou A, Wisnivesky JP, et al. Efficacy of an evidence-based clinical decision support in primary care practices: a randomized clinical trial. JAMA internal medicine. 2013;173(17):1584-91.
13	Persell SD, Lloyd-Jones DM, Friesema EM, Cooper AJ, Baker DW. Electronic health record-based patient identification and individualized mailed outreach for primary cardiovascular disease prevention: a cluster randomized trial. Journal of general internal medicine. 2013;28(4):554-60.
14	Kappen TH, Moons AH, Wolfswinkel Lv, Kalkman CJ, Vergouwe Y, van Klei WA. Impact of Risk Assessments on Prophylactic Antiemetic Prescription and the Incidence of Postoperative Nausea and Vomiting A Cluster-randomized Trial. Anesthesiology. 2014.

Index	Articles
15	Mán E, Simonka Z, Varga A, Rárosi F, Lázár G. Impact of the Alvarado score on the diagnosis of acute appendicitis: comparing clinical judgment, Alvarado score, and a new modified score in suspected appendicitis: a prospective, randomized clinical trial. Surgical endoscopy. 2014;28(8):2398-405.
16	Nieuwlaat R, Eikelboom JW, Schulman S, van Spall HG, Schulze KM, Connolly BJ, et al. Cluster randomized controlled trial of a simple warfarin maintenance dosing algorithm versus usual care among primary care practices. Journal of thrombosis and thrombolysis. 2014;37(4):435-42.
17	Torres FA, Pasarelli I, Cutri A, Ossorio MF, Ferrero F. Impact assessment of a decision rule for using antibiotics in pneumonia: a randomized trial. Pediatric pulmonology. 2014;49(7):701-6.
18[$1]	de Vos-Kerkhof E, Nijman RG, Vergouwe Y, Polinder S, Steyerberg EW, van der Lei J, et al. Impact of a clinical decision model for febrile children at risk for serious bacterial infections at the emergency department: a randomized controlled trial. PloS one. 2015;10(5):e0127620.
19[$2]	Mahler SA, Riley RF, Hiestand BC, Russell GB, Hoekstra JW, Lefebvre CW, et al. The HEART Pathway randomized trial: identifying emergency department patients with acute chest pain for early discharge. Circulation Cardiovascular quality and outcomes. 2015;8(2):195-203.
20	True MW, Strickland LE, Lewi JE, Sterling LM, Dai H, Haas RW, et al. Impact of a Diabetes Risk Score on Lifestyle Education and Patient Adherence (IDEA) in Prediabetes: A Multisite Randomized Controlled Trial. Military medicine. 2015;180(10):1091-7.
21	Zeevi D, Korem T, Zmora N, Israeli D, Rothschild D, Weinberger A, et al. Personalized Nutrition by Prediction of Glycemic Responses. Cell. 2015;163(5):1079-94.

Index	Articles
22	Clemons M, Bouganim N, Smith S, Mazzarello S, Vandermeer L, Segal R, et al. Risk Model-Guided Antiemetic Prophylaxis vs Physician's Choice in Patients Receiving Chemotherapy for Early-Stage Breast Cancer: A Randomized Clinical Trial. JAMA oncology. 2016;2(2):225-31.
23	Guenancia C, Stamboul K, Hachet O, Yameogo V, Garnier F, Gudjoncik A, et al. Clinical effectiveness of the systematic use of the GRACE scoring system (in addition to clinical assessment) for ischaemic outcomes and bleeding complications in the management of NSTEMI compared with clinical assessment alone: a prospective study. Heart and vessels. 2016;31(6):897-906.
24	Sadasivam RS, Borglund EM, Adams R, Marlin BM, Houston TK. Impact of a Collective Intelligence Tailored Messaging System on Smoking Cessation: The Perspect Randomized Experiment. Journal of medical Internet research. 2016;18(11).
25[#, $3]	Sherratt FC, Marcus MW, Robinson J, Field JK. Utilizing Lung Cancer Risk Prediction Models to Promote Smoking Cessation: Two Randomized Controlled Trials. American Journal of Health Promotion. 2016;32(5):1196-205.
26[#, $4]	Steiner JF, Shainline MR, Bishop MC, Xu S. Reducing Missed Primary Care Appointments in a Learning Health System. Medical care. 2016.
27	Steinhart BD, Levy P, Vandenberghe H, Moe G, Yan AT, Cohen A, et al. A Randomized Control Trial Using a Validated Prediction Model for Diagnosing Acute Heart Failure in Undifferentiated Dyspneic Emergency Department Patients-Results of the GASP4Ar Study. Journal of cardiac failure. 2016;23(2):145-52.
28	Allegra A, Marino A, Volpes A, Coffaro F, Scaglione P, Gullo S, et al. A randomized controlled trial investigating the use of a predictive nomogram for the selection of the FSH starting dose in IVF/ICSI cycles. Reproductive biomedicine online. 2017;34(4):429-38.

Index	Articles
29	Brocklehurst P., Field D., Keith Greene, Ed Juszczak, Robert Keith, Sara Kenyon, et al. Computerised interpretation of fetal heart rate during labour (INFANT): a randomised controlled trial. Lancet. 2017;389(10080):1719-29.
30	Caballero-Ruiz E, García-Sáez G, Rigla M, Villaplana M, Pons B, Hernando ME. A web-based clinical decision support system for gestational diabetes: Automatic diet prescription and detection of insulin needs. International journal of medical informatics. 2017;102:35-49.
31[$2]	Poldervaart JM, Reitsma JB, Backus BE, Koffijberg H, Veldkamp RF, Ten Haaf ME, et al. Effect of Using the HEART Score in Patients With Chest Pain in the Emergency Department: A Stepped-Wedge, Cluster Randomized Trial. Ann Intern Med. 2017;166(10):689-97.
32	Shimabukuro DW, Barton CW, Feldman MD, Mataraso SJ, Das R. Effect of a machine learning-based severe sepsis prediction algorithm on patient survival and hospital length of stay: a randomised clinical trial. BMJ Open Respiratory Research. 2017;4(1).
33	K B, A. JH, O. AM. Quality improvement of functional diagnostics in dentistry through computer-aided diagnosis: a randomized controlled trial. International Journal of Computerized Dentistry. 2018.
34	Plomb-Holmes C, Hilfiker R, Leger B, Luthi F. Impact of a non-return-to-work prognostic model (WORRK) on allocation to rehabilitation clinical pathways: A single centre parallel group randomised trial. PloS one. 2018;13(8):e0201687.
35	Snooks H, Bailey-Jones K, Burge-Jones D, Dale J, Davies J, Evans B, et al. Predictive risk stratification model: a randomised stepped-wedge trial in primary care (PRISMATIC). Health Services and Delivery Research. 2018;6(1):1-164.
36	Steiner JF, Shainline MR, Dahlgren JZ, Kroll A, Xu S. Optimizing Number and Timing of Appointment Reminders: A Randomized Trial. Am J Manag Care. 2018.

Index	Articles
37[$5]	Chen D, Wu L, Li Y, Zhang J, Liu J, Huang L, et al. Comparing blind spots of unsedated ultrafine, sedated, and unsedated conventional gastroscopy with and without artificial intelligence: a prospective, single-blind, 3-parallel-group, randomized, single-center trial. Gastrointestinal endoscopy. 2019;91(2):332-9.e3.
38	Cox CE, White DB, Hough CL, Jones DM, Kahn JM, Olsen MK, et al. Effects of a Personalized Web-Based Decision Aid for Surrogate Decision Makers of Patients With Prolonged Mechanical Ventilation. Ann Intern Med. 2019;170(5).
39	Kougias P, Tiwari V, Sharath SE, Garcia A, Pathak A, Chen M, et al. A Statistical Model-driven Surgical Case Scheduling System Improves Multiple Measures of Operative Suite Efficiency: Findings From a Single-center, Randomized Controlled Trial. Annals of surgery. 2019;270(6):1000-4.
40	Lauffenburger JC, Lewey J, Jan S, Makanji S, Ferro CA, Krumme AA, et al. Effectiveness of Targeted Insulin-Adherence Interventions for Glycemic Control Using Predictive Analytics Among Patients With Type 2 Diabetes: A Randomized Clinical Trial. JAMA network open. 2019;2(3):e190657.
41	Lin H, Li R, Liu Z, Chen J, Yang Y, Chen H, et al. Diagnostic Efficacy and Therapeutic Decision-making Capacity of an Artificial Intelligence Platform for Childhood Cataracts in Eye Clinics: A Multicentre Randomized Controlled Trial. EClinicalMedicine. 2019;9:52-9.
42	Palen TE, Sharpe RE, Jr., Shetterly SM, Steiner JF. Randomized Clinical Trial of a Clinical Decision Support Tool for Improving the Appropriateness Scores for Ordering Imaging Studies in Primary and Specialty Care Ambulatory Clinics. AJR Am J Roentgenol. 2019;213(5):1015-20.
43	Su JR, Li Z, Shao XJ, Ji CR, Ji R, Zhou RC, et al. Impact of a real-time automatic quality control system on colorectal polyp and

Index	Articles
	adenoma detection: a prospective randomized controlled study (with videos). Gastrointestinal endoscopy. 2019;91(2):415-24.e4.
44	Thurtle DR, Jenkins V, Pharoah PD, Gnanapragasam VJ. Understanding of prognosis in non-metastatic prostate cancer: a randomised comparative study of clinician estimates measured against the PREDICT prostate prognostic model. Br J Cancer. 2019;121(8):715-8.
45[$6]	Wang P, Berzin TM, Glissen Brown JR, Bharadwaj S, Becq A, Xiao X, et al. Real-time automatic detection system increases colonoscopic polyp and adenoma detection rates: a prospective randomised controlled study. Gut. 2019;68(10):1813-9.
46	Wang SV, Rogers JR, Jin Y, DeiCicchi D, Dejene S, Connors JM, et al. Stepped-wedge randomised trial to evaluate population health intervention designed to increase appropriate anticoagulation in patients with atrial fibrillation. BMJ quality & safety. 2019;28(10):835-42.
47[$5]	Wu L, Zhang J, Zhou W, An P, Shen L, Liu J, et al. Randomised controlled trial of WISENSE, a real-time quality improving system for monitoring blind spots during esophagogastroduodenoscopy. Gut. 2019;68(12):2161-9.
48	Geersing GJ, Hendriksen JMT, Zuithoff NPA, Roes KC, Oudega R, Takada T, et al. Effect of tailoring anticoagulant treatment duration by applying a recurrence risk prediction model in patients with venous thromboembolism compared to usual care: A randomized controlled trial. PLoS medicine. 2020;17(6):e1003142.
49	Gong D, Wu L, Zhang J, Mu G, Shen L, Liu J, et al. Detection of colorectal adenomas with a real-time computer-aided system (ENDOANGEL): a randomised controlled study. The Lancet Gastroenterology & Hepatology. 2020;5(4):352-61.
50	Liu WN, Zhang YY, Bian XQ, Wang LJ, Yang Q, Zhang XD, et al. Study on detection rate of polyps and adenomas in artificial-intelligence-aided colonoscopy. Saudi J Gastroenterol. 2020;26(1):13-9.

Index	Articles
51	Luo Y, Zhang Y, Liu M, Lai Y, Liu P, Wang Z, et al. Artificial Intelligence-Assisted Colonoscopy for Detection of Colon Polyps: a Prospective, Randomized Cohort Study. Journal of gastrointestinal surgery : official journal of the Society for Surgery of the Alimentary Tract. 2020.
52	Mann D, Hess R, McGinn T, Richardson S, Jones S, Palmisano J, et al. Impact of Clinical Decision Support on Antibiotic Prescribing for Acute Respiratory Infections: a Cluster Randomized Implementation Trial. Journal of general internal medicine. 2020;35(Suppl 2):788-95.
53	Manz CR, Parikh RB, Small DS, Evans CN, Chivers C, Regli SH, et al. Effect of Integrating Machine Learning Mortality Estimates With Behavioral Nudges to Clinicians on Serious Illness Conversations Among Patients With Cancer: A Stepped-Wedge Cluster Randomized Clinical Trial. JAMA oncology. 2020;6(12):e204759.
54	Mazurek MO, Parker RA, Chan J, Kuhlthau K, Sohl K. Effectiveness of the Extension for Community Health Outcomes Model as Applied to Primary Care for Autism: A Partial Stepped-Wedge Randomized Clinical Trial. JAMA pediatrics. 2020;174(5):e196306.
55	Meijer F, Honing M, Roor T, Toet S, Calis P, Olofsen E, et al. Reduced postoperative pain using Nociception Level-guided fentanyl dosing during sevoflurane anaesthesia: a randomised controlled trial. British journal of anaesthesia. 2020;125(6):1070-8.
56	Pavel AM, Rennie JM, de Vries LS, Blennow M, Foran A, Shah DK, et al. A machine-learning algorithm for neonatal seizure recognition: a multicentre, randomised, controlled trial. The Lancet Child & adolescent health. 2020;4(10):740-9.
57	Repici A, Badalamenti M, Maselli R, Correale L, Radaelli F, Rondonotti E, et al. Efficacy of Real-Time Computer-Aided Detection of Colorectal Neoplasia in a Randomized Trial. Gastroenterology. 2020.

Index	Articles
58	Tan WJ, Acharyya S, Chew MH, Foo FJ, Chan WH, Wong WK, et al. Randomized control trial comparing an Alvarado Score-based management algorithm and current best practice in the evaluation of suspected appendicitis. World journal of emergency surgery : WJES. 2020;15(1):30.
59[$1]	van de Maat JS, Peeters D, Nieboer D, van Wermeskerken AM, Smit FJ, Noordzij JG, et al. Evaluation of a clinical decision rule to guide antibiotic prescription in children with suspected lower respiratory tract infection in The Netherlands: A stepped-wedge cluster randomised trial. PLoS medicine. 2020;17(1):e1003034.
60	Van Driest SL, Wang L, McLemore MF, Bridges BC, Fleming GM, McGregor TL, et al. Acute kidney injury risk-based screening in pediatric inpatients: a pragmatic randomized trial. Pediatric research. 2020;87(1):118-24.
61[$6]	Wang P, Liu X, Berzin TM, Glissen Brown JR, Liu P, Zhou C, et al. Effect of a deep-learning computer-aided detection system on adenoma detection during colonoscopy (CADe-DB trial): a double-blind randomised study. The Lancet Gastroenterology & Hepatology. 2020;5(4):343-51.
62	Wijnberge M, Geerts BF, Hol L, Lemmers N, Mulder MP, Berge P, et al. Effect of a Machine Learning-Derived Early Warning System for Intraoperative Hypotension vs Standard Care on Depth and Duration of Intraoperative Hypotension During Elective Noncardiac Surgery: The HYPE Randomized Clinical Trial. Jama. 2020.
63	Blomberg SN, Christensen HC, Lippert F, Ersbøll AK, Torp-Petersen C, Sayre MR, et al. Effect of Machine Learning on Dispatcher Recognition of Out-of-Hospital Cardiac Arrest During Calls to Emergency Medical Services: A Randomized Clinical Trial. JAMA network open. 2021;4(1):e2032320.

Notes: Sixty-five randomized controlled trials were identified from 63 clinical studies. Fifty-eight model development studies were found from

the 65 trials. Six pairs of the trials used the same prediction model as intervention, but one pair had no model development study. Thus, a total of 53 prediction models were found.

Articles that included two trials conducted in different population or clinical settings.

$1-$6 Trials used the same prediction model as intervention.

Supplementary Table 3. General characteristics of the observational model development and/or validation studies related to the 65 interventional trials

Variables	Levels	Total (n=65)	TS (n=37)	ML (n=17)	DL (n=11)	P value
Model development (%)	No	7 (10.8)	5 (13.5)	2 (11.8)	0 (0.0)	0.016
	Yes - independent publication	49 (75.4)	30 (81.1)	13 (76.5)	6 (54.5)	
	Yes - published in the same article with RCT	9 (13.8)	2 (5.4)	2 (11.8)	5 (45.5)	
Sample size for model development (n=52, median [IQR])		1392 [198, 10356]	1333 [280, 3542]	50 [17, 3978]	15952 [4115, 88745]	0.004
Unit of sample size (%)	Patient	40 (61.5)	26 (70.3)	11 (64.7)	3 (27.3)	<0.001
	Image or video (colonoscopy/ esophagogastroduodenoscopy)	8 (12.3)	0 (0.0)	0 (0.0)	8 (72.7)	
	Others (cycle/ meal/ ICU stay/ image order)	4 (6.2)	2 (5.4)	2 (11.8)	0 (0.0)	
	Unclear	13 (20.0)	9 (24.3)	4 (23.5)	0 (0.0)	
Publication year (%)	before 2005	8 (12.3)	5 (13.5)	3 (17.6)	0 (0.0)	<0.001
	2006-2010	13 (20.0)	12 (32.4)	1 (5.9)	0 (0.0)	
	2011-2015	16 (24.6)	10 (27.0)	6 (35.3)	0 (0.0)	
	2016-2020	19 (29.2)	4 (10.8)	4 (23.5)	11 (100.0)	

Variables	Levels	Total (n=65)	TS (n=37)	ML (n=17)	DL (n=11)	P value
	Unclear	9 (13.8)	6 (16.2)	3 (17.6)	0 (0.0)	
Study type (%)	Prospective	11 (16.9)	6 (16.2)	5 (29.4)	0 (0.0)	0.016
	Retrospective	41 (63.1)	24 (64.9)	6 (35.3)	11 (100.0)	
	Unclear	13 (20.0)	7 (18.9)	6 (35.3)	0 (0.0)	
Referenced TRIPOD (%)	No	56 (86.2)	31 (83.8)	14 (82.4)	11 (100.0)	0.636
	Yes	2 (3.1)	1 (2.7)	1 (5.9)	0 (0.0)	
	Unclear	7 (10.8)	5 (13.5)	2 (11.8)	0 (0.0)	
Methods of internal validation (%)	Cross-validation	17 (26.2)	7 (18.9)	3 (17.6)	7 (63.6)	0.091
	Bootstrapping	3 (4.6)	3 (8.1)	0 (0.0)	0 (0.0)	
	Random split-sample	7 (10.8)	3 (8.1)	3 (17.6)	1 (9.1)	
	Split-sample by time point	4 (6.2)	2 (5.4)	2 (11.8)	0 (0.0)	
	Unclear	34 (52.3)	22 (59.5)	9 (52.9)	3 (27.3)	
Internal validation (%)	No	23 (35.4)	15 (40.5)	6 (35.3)	2 (18.2)	0.396
	Yes	42 (64.6)	22 (59.5)	11 (64.7)	9 (81.8)	
External validation (%)	No	25 (38.5)	16 (43.2)	7 (41.2)	2 (18.2)	0.313
	Yes	40 (61.5)	21 (56.8)	10 (58.8)	9 (81.8)	

Variables	Levels	Total (n=65)	TS (n=37)	ML (n=17)	DL (n=11)	P value
AUC in model development (n=21, median [IQR])		0.81 [0.75, 0.90]	0.81 [0.76, 0.90]	0.88 [0.74, 0.96]	not reported	0.709
AUC in internal validation (n=18, median [IQR])		0.78 [0.73, 0.88]	0.77 [0.70, 0.84]	0.83 [0.77, 0.88]	0.99 [0.99, 0.99]	0.196
AUC in external validation (n=20, median [IQR])		0.83 [0.79, 0.97]	0.83 [0.76, 0.87]	0.93 [0.93, 0.93]	0.98 [0.98, 0.99]	0.008
Accuracy in model development (n=3, median [IQR])		0.75 [0.72, 0.78]	0.72 [0.71, 0.74]	0.80 [0.80, 0.80]	not reported	0.221
Accuracy in internal validation (n=11, median [IQR])		0.95 [0.93, 0.96]	0.93 [0.93, 0.93]	0.93 [0.89, 0.96]	0.96 [0.94, 0.98]	0.237
Accuracy in external validation (n=8, median [IQR])		0.95 [0.90, 0.98]	0.76 [0.76, 0.76]	0.99 [0.99, 0.99]	0.95 [0.91, 0.98]	0.124

Notes: A total of 58 trials had the model development studies and seven did not have. Missing values of categorical variables were reported as unclear. The available number of continuous data were clarified for each variable. IQR=interquartile range; ICU=intensive care unit.

Supplementary Table 4. Procedures of predictive tool interventions in the seventeen randomized controlled trials involving interventions evaluating machine learning tools

Reference	Conditions	Sample size	Tools for intervention	Control	Algorithms	Tool function	Tool input	Tool output	How the output being used in clinical settings	Trial primary outcomes	Gold standard	Trial findings
Brier 2010	Prevention of anemia for ESRD patients	60	Receiving erythropoietin doses based on the computer recommendations	Receiving erythropoietin doses based on a standard anemia management protocol	Multilayer perceptron network	Assistive treatment decision	Two clinical variables (hemoglobin, previous erythropoietin dose)	Recommendation of erythropoietin dose	Experts validate the safety of recommended erythropoietin dose and then give patients the safe.	Hemoglobin control	Laboratory tests	Positive

Martin 2012	Chronic disease	214	Patient Journey Record system	Usual care	Machine learning and ruled-based algorithms	Assistive treatment decision	Semi-structured conversations about health concerns and well-being	Alerts	Calls were made by lay care guides using the standard pair protocol to generate alerts to trigger local interventions by the clinical participants of the study.	Unplanned acute medical admissions	Experts	Positive

Nieuwlaat 2012	Primary care	1298	Warfarin maintenance dosing algorithm	Simple dosing algorithm already in use in the anticoagulation clinic	Rule-based algorithm	Assistive treatment decision	Weekly warfarin dose and current international normalized ratio result	New weekly warfarin dose	Clinicians referenced the dosing recommendation to make dosing decision and test next international normalized ratio	Time-in-therapeutic range	Laboratory tests	Positive
Finkelstein 2013	Lung transplant recipient home monitoring	65	A computer-based Bayesian triage	Manual nurse triage	Bayesian algorithm	Assistive treatment decisio	Total reported severity of six different symptoms,	Probability of event of meaningful increases in	Patients for whom the probability of event is	Change from baseline after 1 year in	Laboratory tests	Negative

			algorithm			n	FEV1, age, gender	symptoms and/or drops in log(FEV1) ratio	high are contacted to set up physician visits	forced expiratory volume at 1s FEV1		
Hsu 2013	ICU weaning	312	Patients were weaned with CDSS monitored by physicians	Patients were weaned with traditional weaning method	Support vector machine	CDSS	Eleven variables, including demographic information, physiology and disease factors, and care and treatment factors for CDSS	Successful weaning probability; Suggestion to wean the mechanic ventilator from the patient or not	Physicians made decision of weaning for patients with the assistance of CDSS	The sensitivity of weaning successfully	Clinical criteria	Positive

Nieuwlaat 2014	Primary care	537	Warfarin maintenance dosing algorithm	Usual care	Rule-based algorithm	Assistive treatment decision	Weekly warfarin dose and current international normalized ratio result	Recommended following dose adjustments; specific advice for the international normalized ratio retest	Clinicians referenced the dosing recommendation to make dosing decision	Time-in-therapeutic range	Laboratory tests	Negative
Zeevi 2015	Prediabetics	26	Prediction arm using a personalized diet algorithm	Expert arm	Gradient boosting regression	Assistive treatment decision	Microbiome; blood tests; questionnaires; anthropometrics; food diary	Rankings of every meal for each participant	Clinicians used the rankings generated by the algorithm to design	Postmeal glycemic responses	Experts	Positive

									two 1-week diet.			
Sadasivam 2016	Smoking	120	A standard rule-based computer-tailored health communication system with receiving motivational messages tailored by the perspect recommen	A standard rule-based computer-tailored health communication system	Innovative hybrid machine learning recommender system	Assistive or change	User profile information and the metadata description of the item or message	Motivational messages	Motivational messages were sent to smokers.	The proportion of days when smokers agreed/strongly agreed that the messages influenced them to quit	Subjectsdecision	Positive

			der system									
Brocklehurst 2017	Fetal heart-rate monitoring	47062	Decision support with the INFANT system in conjunction with cardiotocography	Cardiotocography with no decision support	Numerical algorithms and a small neural network	CDSS	Fetal heart signals	Colour-coded alert	The decision-support software does not provide recommendations for any action that should be taken in response to abnormalities. All clinicians	Poor neonatal outcome	Clinical data	Negative

								supervising labour are expected to have been trained in the appropriate response to an abnormal cardiotocographic reading			
Caballero-ruiz 2017	Gestational diabetes	90	Remote patients' monitoring using	Telemedical care process with	Expectation maximization	CDSS	Glycaemia data at home; ketonuria; compliance	Therapy adjustment recommendations;	Diet recommendations are automatica	Physician-patient interaction, etc. No	Positive

SINEDIE, a web-based telemedicine CDSS	conventional care	clustering algorithm and a c4.5 decision tree learning algorithm		to dietary treatment	diet recommendations; recommendations about insulin requirements	lly prescribed and notified to the patients, whereas recommendations about insulin requirements are notified also to the physicians, who will decide if insulin	primary outcome was prespecified.

needs to be prescribed. The medical team followed the patients' treatment with the SINEDIE system until the insulin therapy was needed or the pregnancy

								ended.			
Shimabukuro 2017	Severe sepsis	142	Current severe sepsis detector with the assistance of machine learning algorithm	Current severe sepsis detector	Machine learning algorithm	Assistive treatment decision	Four patient variables (vitals, peripheral capillary oxygen saturation, Glasgow Coma Score, and age)	Alerts	On receiving an alert, the care team evaluated the patient and initiated the severe sepsis bundle.	Average hospital length of stay	Medical records	Positive
Becker 2018	Craniomandibular disorders and	390	Computer-aided diagnosis (cmdfact 4	Traditional diagnostic method	Computer-aided diagnosis	Assistive diagnosis	Patients' findings from the clinical	Possible diagnoses; grading; weighting	Examiners referenced computer output and	Diagnosis agreement of a	Principle investigators provide	Positive

	temporomandibular disorders		functional diagnostics software)				functional analysis and manual structural analysis		give their diagnosis	series outcome		
Plomb-holmes 2018	Vocational inpatients working disability assessment after orthopaedic trauma	280	Estimating non-return to work risk using the prognostic model and sending to clinicians	Estimating non-return to work risk using the prognostic model but not sending to clinicians	Random forest	Risk stratification	Nineteen factors (1 occupational, 6 biomedical, and 12 psychosocial factors)	The non-return to work risk with three pathways (simple, coaching and evaluation); indications for interpretation of the	The risk was given to the clinician team before allocation of vocational pathways.	Proportion of patients allocated to the evaluation pathway	The clinical decision of patients allocation to the evaluation pathway	Negative

								risk				
Wang S 2019	Atrial fibrillation (AF) at high risk of stroke	1727	Complex intervention	Usual care	Machine learning algorithm	Risk stratification	Several risk assessment model	Algorithm-identified high-risk patients with AF in clinics (CHA2,DS2,-vasc risk score ≥2)	The intervention involved (1) applying algorithms to efficiently identify relevant patients from the EHR, (2) secondary chart review of algorithm-	The proportion of algorithm-identified eligible patients prescribed an oral anticoagulants in the 28 days after entering the interventi	Experts	Negative

identified patients by clinical staff, (3) an offer to primary care providers (PCP) to assist with anticoagulation management from an established clinical service, and (4) targeted	on or ‘usual care’ arm at each step in the stepped wedge

									reminders from clinical leaders.			
Manz 2020	Serious illness conversations (SICs)	14607	Clinician-directed intervention integrating machine learning mortality predictions with behavioral nudges on motivating clinician-patient	Clinicians received usual care consisting of weekly emails with cumulative SIC performance	Gradient boosting algorithm	Assistive treatment decision	Structured electronic health record data	(1) Weekly emails to oncology clinicians with SIC performance feedback and peer comparisons; (2) a list of up to 6 high-risk patients scheduled for the next	Clinicians referenced the output to make decision of SIC or not.	Percentage of patient encounters with an SIC	The clinical decision of SICs	Positive

SICs	week, estimated using a validated machine learning algorithm; and (3) opt-out text message prompts to clinicians on the patient's appointment day to consider an SIC.

Meijer 2020	Postoperative pain	50	Nociception Level (NOL) monitor, a multiparameter AI-driven index - guided opioid dosing	Standard care that fentanyl dosing dependent solely on haemodynamics	Random forest	Assistive treatment decision	Five factors (finger photoplethysmogram amplitude, skin conductance, Heart Rate, Heart Rate variability, and their time derivatives)	NOL index	Fentanyl dosing was dependent on the NOL index	Postoperative pain scores	Clinical criteria	Positive
Pavel 2020	Neonatal seizures	258	cEEG plus Algorithm for Neonatal Seizure Recognitio	cEEG monitoring alone	Support vector machine	Assistive diagnosis	Fifty-five features extracted from EEG recordings	A seizure probability trend in real time; alarm and red marker	Clinical team referenced algorithm output and make	Diagnostic accuracy (sensitivity, specificit	Experts	Positive

	n linked to the EEG monitor	diagnosis	y, and false detection rate) of health-care professionals to identify neonates with electrographic seizures

Abbreviations: EEG = electroencephalography; ICU = Intensive care unit.
